# Superficial vimentin mediates DENV-2 infection of vascular endothelial cells

**DOI:** 10.1038/srep38372

**Published:** 2016-12-02

**Authors:** Jie Yang, Lingyun Zou, Yi Yang, Jizhen Yuan, Zhen Hu, Hui Liu, Huagang Peng, Weilong Shang, Xiaopeng Zhang, Junmin Zhu, Xiancai Rao

**Affiliations:** 1 Department of Microbiology, College of Basic Medical Sciences, Third Military Medical University, Key Laboratory of Microbial Engineering under the Educational Committee in Chongqing, Chongqing 400038, People’s Republic of China

## Abstract

Damage to vascular endothelial cells (VECs) is a critical hallmark of hemorrhagic diseases caused by dengue virus (DENV). However, the precise molecular event involved in DENV binding and infection of VECs has yet to be clarified. In this study, vimentin (55 kDa) was identified to be involved in DENV-2 adsorption into VECs. This protein is located on the surface of VECs and interacts with DENV-2 envelope protein domain III (EDIII). The expression level of the superficial vimentin on VECs was not affected by viral infection or siRNA interference, indicating that the protein exists in a particular mode. Furthermore, the rod domain of the vimentin protein mainly functions in DENV-2 adsorption into VECs. Molecular docking results predicted several residues in vimentin rod and DENV EDIII; these residues may be responsible for cell–virus interactions. We propose that the superficial vimentin could be a novel molecule involved in DENV binding and infection of VECs. DENV EDIII directly interacts with the rod domain of vimentin on the VEC surface and thus mediates the infection.

Dengue virus (DENV) causes more than 200 million infections per year and affects approximately 3.6 billion people worldwide^1^. DENV is an important public concern because it seriously threatens public health. Diseases caused by DENV vary from mild-to-debilitating febrile illnesses (classical dengue fever) to fatal syndromes (dengue hemorrhagic fever/dengue shock syndrome, DHF/DSS); the mortality rate of such diseases ranges from 5% to 30%^2^,^3^,^4^. The hallmark characteristics of DHF/DSS are unbalanced homeostasis and increased vascular permeability; the pathogenesis of the disease was speculated to be the dysfunction of vascular endothelial cells (VECs)^5^,^6^. VECs are the main target cells directly or indirectly affected during DENV infection. However, the precise molecular events and the specific receptor(s) or causative agent(s) involved in DENV binding and infection of VECs must be further elucidated.

Studies on the interaction between DENV and VECs *in vivo* are restricted by technical difficulties and shortage of suitable animal models; as such, scholars commonly used cell lines derived from primary human VECs and VECs; these cell lines include human umbilical vein ECs (HUVECs), human microvascular ECs (HMEC-1 cells), liver sinusoidal ECs (LSECs), human pulmonary ECs (HPMEC-ST1.6 R cells), and endothelial cells (ECV304 cells)^7^,^8^,^9^,^10^,^11^,^12^,^13^. Despite that many cell receptors or corresponding factors for DENV infection of VECs have been identified using cell-infection models, only several molecules of interest are reportedly involved in DENV adsorption. Zhang *et al*.^8^ showed that DENV-2 infection upregulated the expression of β3 integrin in HMEC-1 cells; moreover, pre-incubation of DENV-2 with soluble ανβ3 integrin inhibits viral entry. However, antibodies against ανβ3 integrin do not completely block the DENV infection^8^, indicating that additional co-factors may be involved in the process. Dalrymple and Mackow reported that pretreatment of human ECs with heparin or heparan sulfate could reduce dengue infection by 60% to 80%^12^; this finding suggests that DENV could attach to heparan sulfate-containing proteoglycan receptors on ECs. Furthermore, other receptors or co-factors may be involved in DENV infection. In our previous study, we identified 34, 43, and 55 kDa membrane proteins of ECV304 cells as potential host factors for DENV infection^14^. Another study identified the two former proteins (34 and 43 kDa)^15^. The 43 kDa protein, which was identified as actin, is involved in DENV infection of ECV304 cells^14^. Considering these findings, we aim to determine the function of the novel 55 kDa protein in DENV infection.

In this study, the 55 kDa protein was identified as vimentin and was found to be expressed on the surface of VECs. This type of vimentin (superficial) existed in a particular mode on the surface of VECs. The expression of the protein was not affected by DENV infection and siRNA interference. The vimentin protein possesses three structural domains; of which, the rod domain is mainly involved in DENV adsorption. Several residues, with a docking complex of vimentin and DENV-2 EDIII, were predicted to be located in both vimentin rod domain and DENV EDIII and may be responsible for host–virus interactions.

## Results

### Vimentin, a 55 kDa protein, interacts with DENV-2 EDIII

**Figure 1:**
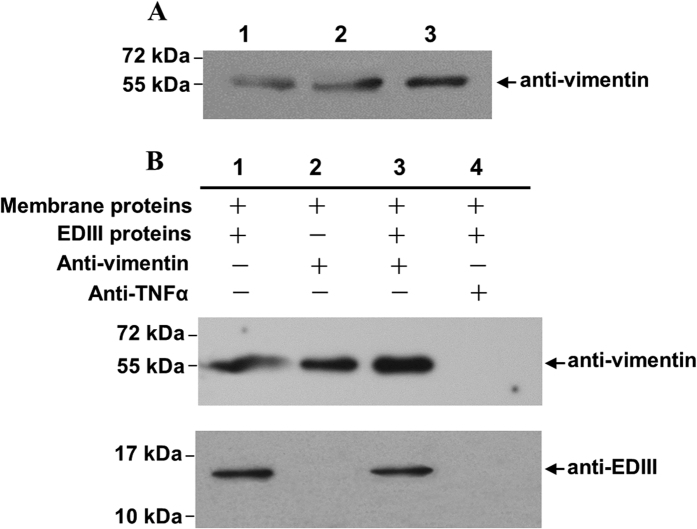
Characterization of the 55 kDa protein derived from the plasma membrane proteins of ECV304 cells. (**A**) Western blot analysis of the 55 kDa protein. The total ECV304 cell proteins (lane 1), ECV304 cell plasma membrane proteins (lane 2), and the 55 kDa proteins (lane 3) excised from an SDS-PAGE gel of the plasma membrane proteins of ECV304 cells were separated by 12% SDS-PAGE and analyzed with commercial rabbit anti-vimentin PcAb as described in Materials and Methods. Protein sizes are indicated on the left. (B) Co-immunoprecipitation (Co-IP) experiment. The mixture of the ECV304 cell plasma membrane proteins and the purified DENV-2 EDIII was either directly analyzed (lane 1, served as protein controls) or analyzed after Co-IP assay with commercial rabbit anti-vimentin PcAb (upper panel) or mouse anti-DENV-2 EDIII PcAb (bottom panel) as indicated by an arrow on the right for testing the presence of proteins of interest (lane 3). Co-IP without DENV-2 EDIII proteins (lane 2) or with an isotype Ab (anti-TNFα, lane 4) served as controls. The full-length blots are presented in Supplementary Figure 1.

Virus overlay protein binding assay (VOPBA) is widely used to screen virus-associated cell factors^14^,^15^. A study that used the modified assay reported that the 55 kDa membrane protein derived from ECV304 cells interacted with DENV-2 EDIII^14^. To characterize this protein, the relative band was excised from the Coomassie blue-stained gel [12% sodium dodecyl sulfate-polyacrylamide gel electrophoresis (SDS-PAGE)] and detected by high-performance liquid chromatography (HPLC)–(micro)chip–mass spectrometry (MS)/MS (Agilent Technologies, CA) analyses. Mass data were searched against the IPI human database of peptide masses (http:// www.ebi.ac.uk/IPI). A family of strong peaks, corresponding to the fragmentation of vimentin–*Homo sapiens* (human), was identified. Western blot analysis was conducted to test the authenticity of vimentin, and confirmation was performed using a commercial mouse anti-vimentin polyclonal antibody (PcAb) as probe (Fig. 1A). The result indicates that the 55 kDa protein is vimentin.

Interaction between DENV-2 EDIII and 55 kDa vimentin was tested by co-immunoprecipitation (co-IP) assay. DENV-2 EDIII co-immunoprecipitated with vimentin (Fig. 1B, lane 3), and the complex was more evident than that in DENV-2 EDIII-containing mixture (Fig. 1B, lane 1) but was not found in the blank control (no EDIII protein, Fig. 1B, lane 2) and isotype Ab control (anti-TNFα, Fig. 1B, lane 4). Collectively, the 55 kDa vimentin derived from ECV304 membrane proteins interacted with DENV-2 EDIII, implying the crucial function of this protein in DENV infection.

### Superficial vimentin of human VECs shows high co-localization with DENV-2

**Figure 2:**
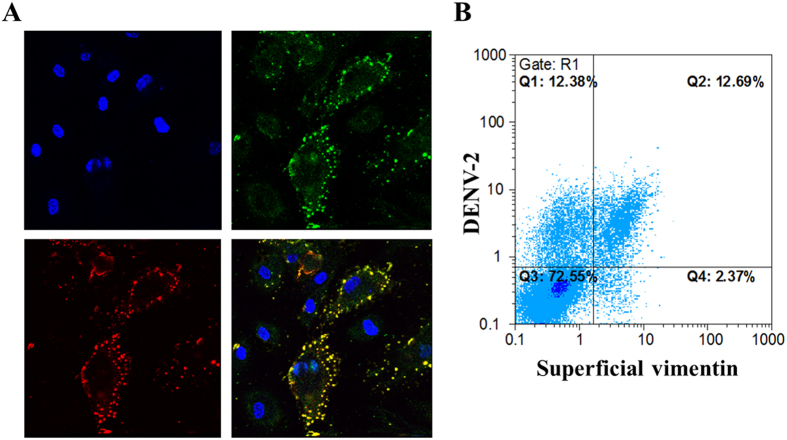
DENV-2 showed high co-localization with the superficial vimentin on VECs. (**A**) Confocal laser microscopy of DENV-2 and the superficial vimentin on VEC surface. HUVEC nucleus stained by DAPI (upper left panel), DENV-2 adsorbed on HUVECs surface detected by mouse anti-DENV-2 EDIII PcAb at 4 °C (upper right panel), the superficial form of vimentin on HUVECs detected by anti-vimentin PcAb without permeability at 4 °C (bottom left panel), and the merged image (bottom right panel) (magnification, ×1,000). (**B**) Flow cytometric analysis of the expression level of superficial vimentin on ECV304 cells and the binding DENV-2 particles.

Vimentin is a main cytoskeletal protein that maintains the cytoplasm architecture. Cytoplasmic vimentin interacts with viruses during virion assembly and facilitates virion transportation^16^,^17^. Moreover, vimentin can be secreted by activated cells^17^. However, the function of secreted vimentin in virus infection remains unknown. Considering the interaction between DENV-2 EDIII and plasmalemmal vimentin of ECV304 cells, we aim to determine whether DENV-2 can interact with vimentin on the surface of VECs. ECV304 cells are considered as endothelial-like cells, whereas HUVECs (primary human umbilical VECs) can imitate DENV infection *in vivo.*^12^,^13^ To observe the molecular events on the cell surface, we infected HUVECs with DENV-2 on ice or at 4 °C. The molecular events were determined using PcAbs against DENV-2 EDIII and vimentin. Confocal microscopy findings indicated that DENV-2 was absorbed onto the surface of HUVECs, and vimentin was expressed on the cell surface (Fig. 2A); these findings are consistent with those reported in previous studies^13^,^18^. DENV-2 showed high co-localization with superficial vimentin on HUVECs in the viral adsorption stage; however, a proportion of HUVECs did not contain superficial vimentin (Fig. 2A). For easy observation, we used ECV304 cells in the experiments requiring relatively high expression level of superficial vimentin. Based on flow cytometric analysis, the superficially expressed vimentin on ECV304 cells was correlated with the binding of DENV-2 particles (Fig. 2B); 12.69% of the total population was presented as DENV-positive cells. However, approximately half of DENV-positive ECV304 cells (12.38%) showed low expression level of superficial vimentin. This finding indicates that other factors (such as β3 integrin) may be involved in DENV binding to VECs, and vimentin only acts as a major co-receptor or binding molecule in the process.

### Expression of superficial vimentin is not affected by DENV-2 infection or siRNA interference

**Figure 3:**
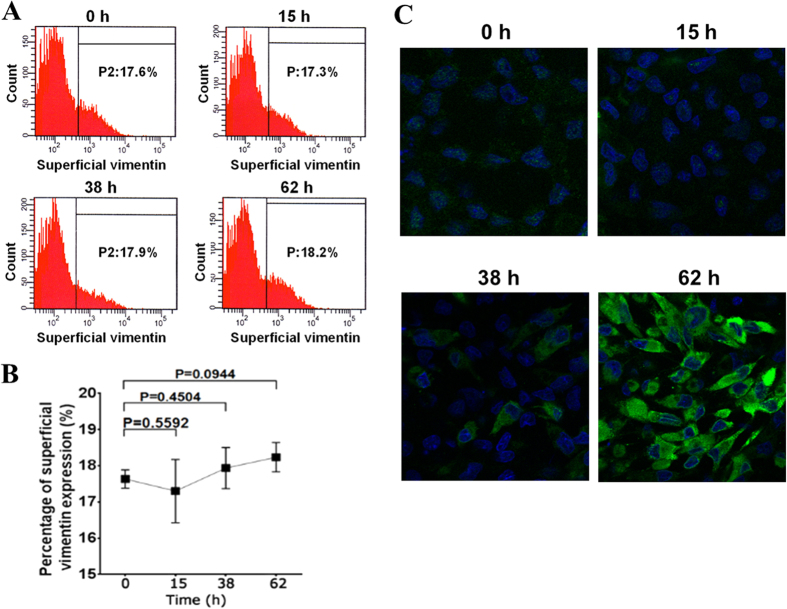
Expression level of superficial vimentin unaffected by DENV-2 infection. (**A**) Flow cytometric analysis of superficial vimentin on DENV2-infected ECV304 cells at 15, 38, and 62 h post-infection (hpi), and 0 h infected cells were used as control. (**B**) Quantitative analysis of superficial vimentin on DENV2-infected ECV304 cells at the indicated time points. Data are presented as means ± standard deviation (S.D.) (*n* = 3). *P-*values were indicated versus 0 h infection. (**C**) IFA of DENV-2 infected ECV304 cells at the indicated time points. The DENV-2 infected ECV304 cells were detected with the mouse anti-DENV-2 EDIII pcAb, and cell nuclei were stained by DAPI as described in Materials and Methods. After washing, the cells were observed under a confocal laser microscope (magnification, ×400).

**Figure 4:**
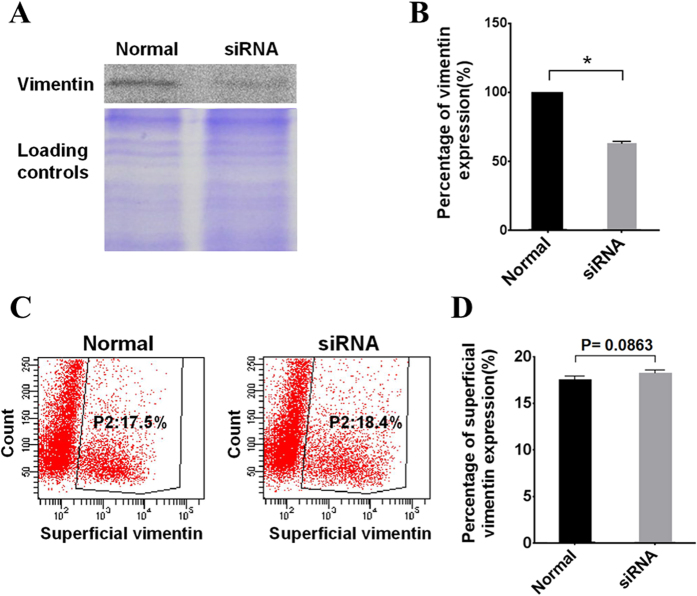
Expression level of the superficial vimentin unaffected by siRNA interference. (**A**) Western blot analysis shows the knockdown efficiency of total vimentin in transfected ECV304 cells with siRNA targeting vimentin compared with untransfected normal cells. Equal loading of the gels was confirmed by staining the gel with Coomassie brilliant blue G-250. The full-length blots/gels are presented in Supplementary Figure 2. (**B**) The protein gray value was relative to protein expression level. The total vimentin protein gray values of normal ECV304 cells and siRNA transfected cells were analyzed by Image-Pro Plus 6.0 software. The percentage of vimentin expression after siRNA interference was plotted in comparison with that in normal cells, which was set to 100%. The * indicates *P < *0.01. (**C**) ECV304 cells were transfected with vimentin siRNA; after 48 h, the cell superficial vimentin was tested by flow cytometric analysis. Untransfected normal cells were used as negative control. (**D**) Quantitative data of flow cytometric analysis are presented as means ± S.D. (*n* = 3), and the *P-*value was indicated.

The expression level of β3 integrin increases during DENV infection^8^. In this study, flow cytometry was performed to test the effect of DENV-2 infection to the expression level of superficial vimentin. Surprisingly, no significant change in the expression level of superficial vimentin on VECs was observed at 15, 38, and 62 h post-DENV-2 infection (hpi) compared with that at 0 h of infection (Fig. 3A,B). DENV-2 infection gradually occurred in ECV304 cells at 15, 38, and 62 hpi and was detected with indirect immunofluorescence assay (IFA, Fig. 3C). However, the pathway underlying the secretion for superficial vimentin in VECs remains unknown. We then investigated the effect of knockdown of vimentin expression in VECs on the amount of superficial vimentin on cells by using siRNA interference. ECV304 cells transient transfected with commercial siRNA targeting vimentin (Santa Cruz Biotechnology, CA) were collected. Total vimentin was determined by Western blot analysis. Superficial vimentin was detected by flow cytometry analysis. As shown in Fig. 4A,B, the total cellular vimentin was significantly downregulated by specific siRNA interference, whereas the superficial vimentin remained constant (Fig. 4C,D). These results suggest that the expression mode of the superficial vimentin on VECs is not similar to that of β3 integrin, which could be upregulated by viral infection^8^.

### Rod domain of vimentin mainly functions in DENV-2 binding and adsorption

**Figure 5:**
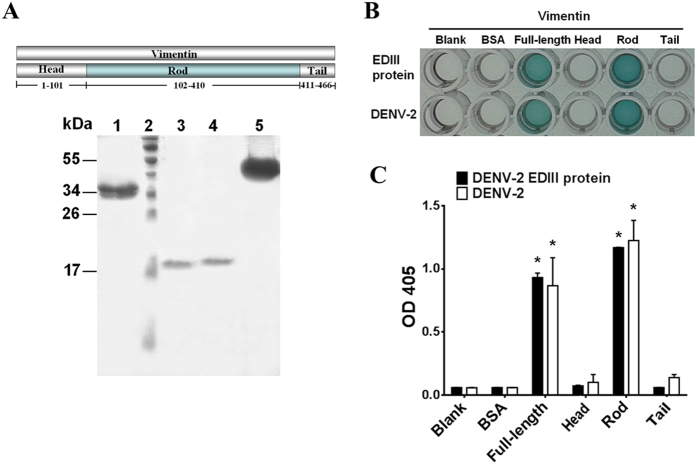
Purified vimentin rod protein specifically binds to DENV-2 EDIII. (**A**) Purified vimentin proteins of the rod (a.a. 102–410) (Lane 1), tail (a.a. 411–466) (Lane 3), and head (a.a. 1–101) (Lane 4) domains, and the full-length vimentin (Lane 5) were analyzed by SDS-PAGE. Protein sizes were indicated on the left. (**B**) Vimentin–binding ELISA. The binding levels of vimentin head, rod, and tail domain proteins, and the full-length protein with DENV-2 or DENV-2 EDIII protein, respectively, showed that both DENV-2 EDIII protein and DENV-2 could specifically bind to vimentin rod and the full-length vimentin proteins. BSA served as negative control and PBS served as blank control. (**C**) Protein-binding levels were represented by reads of the ELISA plates at 405 nm on a VERSAmax tunable microplate reader. All experiments were repeated in triplicate and reported as average and S.D.; *P-*values were indicated versus BSA negative control. * indicates *P < *0.01.

Vimentin, as the major component of the cytoskeleton, consists of a head domain (amino acids, a.a. 1–101), a central rod domain (a.a. 102–410), and a tail domain (a.a. 411–466)^16^,^19^. To find the key domain donated to DENV adsorption, we engineered three truncated proteins corresponding to the three domains of vimentin and the full-length vimentin proteins to be expressed and purified (Fig. 5A). An enzyme-linked immunosorbent assay (ELISA) was performed to test the binding activity of each segment of vimentin to DENV-2 EDIII and DENV-2. Only the rod domain and full-length vimentin could bind to the DENV-2 EDIII protein and whole DENV-2 particles (Fig. 5B,C).

**Figure 6:**
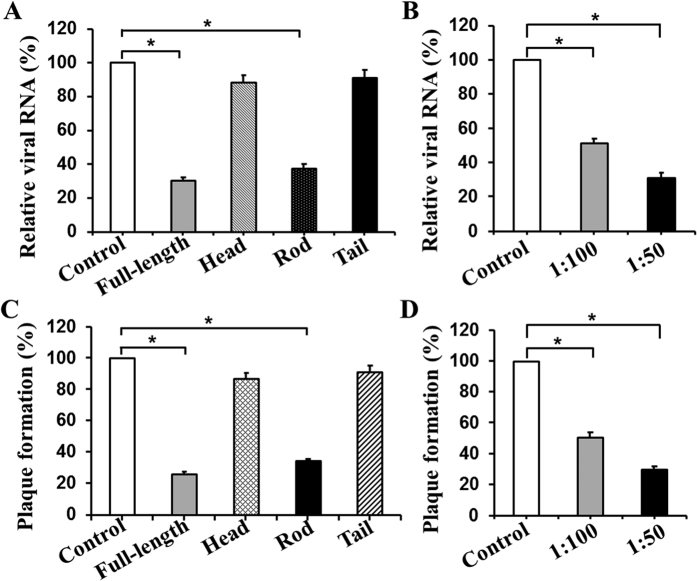
Rod domain of vimentin plays a key role in DENV-2 infection of HUVECs determined by protein and the antibody blocking assays. (**A**) qRT-PCR determination of viral RNA in HUVECs infected with vimentin protein-incubated DENV-2. DENV-2 was pre-incubated with four purified vimentin proteins as indicated at 4 °C for 1 h. BSA was used as negative control. Virus–protein complex was then added to the HUVEC monolayer at 37 °C for 2 h. After washing with PBS, the cells were collected for total RNA extraction and qRT-PCR determination as described in Materials and Methods. The relative expression levels of viral RNA were normalized to HUEL housekeeping gene, and the BSA control was set to 100%. (**B**) qRT-PCR determination of viral RNA in DENV-2 infected HUVECs pre-blocked with PcAb. HUVECs were pre-incubated with anti-vimentin rod PcAb V4630 at dilutions of 1:50 and 1:100 at 4 °C for 1 h. Normal mouse sera (1:100) were served as control. The cells were then infected with DENV-2 (MOI = 1) at 37 °C for 2 h. After washing with PBS, the cells were collected for total RNA extraction and qRT-PCR determination as described in Materials and Methods. (**C**) Plaque forming experiment after protein blocking. The blocking assay with vimentin proteins and DENV-2 infection of HUVECs were performed as described. The intracellular virus titer was detected by plaque forming assay using Vero cells. (**D**) Plaque forming experiment after antibody blocking. The entry of DENV-2 is effectively blocked by anti-vimentin rod PcAb V4630 in a dose-dependent manner. All data are presented as means ± S.D. (*n* = 3). * represented *P* < 0.05 versus control.

If the binding of vimentin rod domain to DENV-2 EDIII mediates the DENV infection of VECs, then the viral infection could be inhibited. The inhibition could occur when DENVs were pre-incubated with the vimentin rod proteins or when HUVECs were pre-treated with V4630 PcAb against vimentin rod prior to viral infection. The viral RNA levels were determined by quantitative real-time RT-PCR (qRT-PCR) analysis for the NS1 gene; the levels significantly decreased in DENV-2 infected HUVECs post full-length vimentin and vimentin rod protein incubations (Fig. 6A) or V4630 PcAb blocking (Fig. 6B). The viral RNA levels significantly decreased compared with those in control cells. A plaque forming experiment was performed to further confirm these findings. The experiment revealed that approximately 74.0% and 65.7% of plaque reductions were observed for DENV-2 infectivity in the blocking experiments with full-length vimentin and vimentin rod proteins compared with control. The viral infection was only blocked by 13.2% and 8.9% of plaque reductions with vimentin head and tail proteins, respectively (Fig. 6C). PcAb V4630 could effectively block DENV-2 entry into HUVECs in a dose-dependent manner. The 100-fold dilution of V4630 could reduce approximately half of DENV-2 infection, and the 50-fold dilution of the antibody could even block up to 70% of the infection (Fig. 6D). Thus, the vimentin rod is the key domain that interacts with DENV EDIII. This interaction serves an important function in the course of DENV infection to VECs.

### Prediction of residues involved in the interaction between DENV-2 EDIII and vimentin rod domain

**Figure 7:**
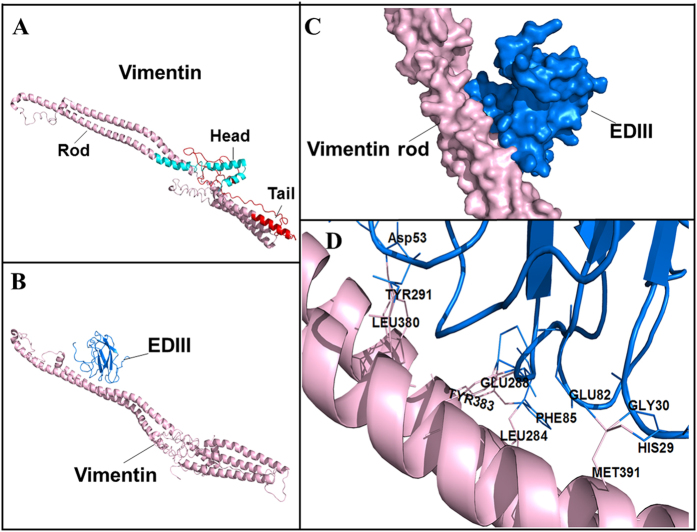
Prediction of residues involved in the interaction between DENV-2 EDIII and vimentin rod. (**A**) 3D structure of the full-length vimentin made by MODELLER (head, rod, and tail domains appear red, pink, and green, respectively). (**B**) Docked model of vimentin rod (pink) and DENV-2 EDIII protein (blue) established by ZDOCK V3.0.2 software (shown as cartoons). (**C**) The docked model (shown as protein surfaces) to show the overall interaction. (**D**) Prediction of functional residues involved in the binding between vimentin rod and DENV-2 EDIII by the InterproSurf software and interacting interface analysis by the Pymol 1.7.3 software. The relative amino acids are labeled as indicated.

Precise identification of residues involved in the interaction between DENV-2 EDIII and vimentin rod is complex; as such, the structure of the full-length vimentin was modeled (Fig. 7A), and a docking model was established for the vimentin–DENV-2 EDIII complex (Fig. 7B). Consistent with the experimental results, EDIII tended to interact with the rod domain of vimentin (Fig. 7C). Five residues (Asp53, Phe85, Glu82, Gly30, and His29) of DENV-2 EDIII and six residues (Tyr291, Leu380, Glu288, Tyr383, Leu284, and Met391) on the vimentin rod were predicted as hot sites involved in the interaction of the two proteins (Fig. 7D). The actual roles of these predicted residues in the interaction between DENV-2 EDIII and vimentin rod should be further investigated.

## Discussion

The endothelium is the ultimate target for DENV infection. Once infected with DENV, VECs produce abundant proinflammatory cytokines, including IL-8, resulting in capillary leakage and endothelial cell barrier dysfunction, which are typical pathological basis of DHF and DSS^11^. A study on the interaction between DENV and VECs explains the pathogenesis of DENV-caused diseases and provides a theoretical basis for prophylaxis of dengue infection.

The initial step of viral infection is the attachment of a virus to receptors to the cell surface. The attachment mode varies among viruses. Certain viruses bind to a single receptor to enter host cells; other viruses use more than one receptor for a single cell or different host cells, and some viruses require additional co-receptors or host factors for entry^20^,^21^,^22^,^23^. The exact molecular events for virus–receptor attachment and corresponding receptors for DENV infection must be clarified. Several proteins, such as β3 integrin, heparin or heparan sulfate, protein disulfide isomerase, and 43 kDa protein, were partly involved in DENV infection of VECs^8^,^12^,^14^,^15^,^24^; these findings indicate that DENV may use more than one receptor or co-receptor to infect VECs.

In our previous study, we identified a new 55 kDa membrane protein involved in DENV infection of VECs; the protein was identified as vimentin, an important cytoskeletal protein in mesenchymal cells^25^,^26^. Vimentin, which belongs to class III intermediate filaments, is located in the cytoplasm and functions as an intracellular scaffold that maintains the structural and mechanical integrity of a cell^27^. Nevertheless, several studies confirmed that vimentin is indispensable for viral infection. Some viruses can interact with vimentin or modify its cytoskeleton behavior in host cells, resulting in virus entry, intracellular transportation, maturation, and release^28^,^29^,^30^,^31^. In our previous study, we revealed that intracellular vimentin is required for DENV infection^32^. The binding of DENV with its receptor(s) could induce Rho-associated coiled coil-containing kinase (ROCK) activation, leading to intracellular vimentin rearrangement and endoplasmic reticulum redistribution around the perinuclear region; these processes facilitated the anchor of DENV to the replication niche and formation of viral factory^32^. Vimentin reorganization might be important in DENV infection. Vimentin proteins retracted from the cell periphery, surrounded the nucleus in DENV-2 infected cells, and colocalized with DENV-2 antigens. However, the fluorescence intensity of DENV-2 antigens decreases, and the colocalization of DENV-2 antigens with vimentin cannot be observed after the disruption of vimentin with acrylamide^33^.

Vimentin is found on the surface of numerous cells, such as apoptotic neutrophils and T cells^34^,^35^,^36^, Sezary T cells^37^, activated macrophages^38^, VECs^25^, skeletal muscle cells, brain microvascular ECs^39^, and platelets^40^. However, the mechanisms of this protein remain unknown. Thus, the function of superficial vimentin in infection process of a pathogen must be investigated. We found that DENV-2 could infect VECs by directly interacting with the rod domain of the superficial vimentin. Other viruses, such as porcine reproductive respiratory syndrome virus and Japanese encephalitis virus (JEV), can also target superficial vimentin^19^,^28^,^31^. Therefore, superficial vimentin may be a versatile host factor for viral infection, but the exact binding sites in vimentin may vary in different species of viruses.

The expression mode of superficial vimentin may also vary in cells or microbial infections. Garg *et al*.^17^ reported that the infection of monocytes with *Mycobacterium tuberculosis* increases superficial vimentin; the vimentin then acts as a ligand for NKp46 receptor on natural killer cells. However, the expression of superficial vimentin on VECs seems to be constitutive. We showed that the amounts of superficial vimentin on VECs are unaffected by DENV-2 infection and knockdown of vimentin via siRNA interference. Xu *et al*.^25^ showed that the secreted form of vimentin does not originate from an endothelial cell-specific mRNA transcript, but is the product of cell-specific posttranslational modification. The mechanisms underlying vimentin modification and presentation on VECs are of interest and should be further investigated.

We also revealed that the rod domain of vimentin serves a key function in DENV-2 binding and adsorption to VECs. However, PcAb specific against vimentin rod could not completely block DENV-2 entry into VECs. Almost half of DENV-infected VECs showed low expression of superficial vimentin. These findings indicate that additional co-factors may exist and are involved in the entry process of DENV into VECs. This result agrees with that of a previous study, which showed that ανβ3 integrin serves a substantial function in DENV infection, and the antibodies against ανβ3 integrin do not completely block the infection^8^. The usage of different host factors for virus adsorption complicated the understanding of DENV pathogenesis, thereby explaining the wide cell tropism of DENV in nature.

Endothelial permeability was ultimately changed by VECs lining of the vasculature, as well as DENV-induced responses resulting in hemorrhagic disease such as DHF and DSS^12^. Blood flow has a strong force to the blood vessel wall, and DENV must have a strong adsorption mechanism to contact the surface of VECs for entry. Flavivirus variants with enhanced glycosaminoglycans (GAGs) binding capacity are quickly cleared from the bloodstream^19^. Liang *et al*.^19^ demonstrated that the attenuated variant of JEV prefers to bind GAGs on host cells, whereas the virulent JEV prefers to bind superficial vimentin to overcome the clearance of blood flow; this finding suggested that vimentin binding is critical for virulent JEV infection. In the present study, we revealed the crucial roles of superficial vimentin for DENV adsorption to VECs. We propose that the superficial vimentin acts as a recruiting device that increases the infection of DENV to VECs. To fully understand the mechanism of DENV infection, further studies should focus on whether ανβ3 integrin and superficial vimentin can simultaneously work to participate in the DENV entry of VECs, as well as the situation where the two factors cooperate.

In conclusion, we reveal for the first time that the superficial form of vimentin on VECs serves an important function in DENV infection. Superficial vimentin may serve as a co-receptor, interacts with EDIII protein of DENV, facilitates DENV adsorption to VECs, and overcomes bloodstream wash-out in vasculature. However, the actual existence mode of vimentin on VECs surface and the exact sites involved in the adsorption of DENV to VECs require further investigations.

## Materials and Methods

### Cell lines and viruses

Vero African green monkey kidney cells and ECV304 cells (ATCC CRL 1998) were maintained in Dulbecco’s modified eagle medium (DMEM; HyClone, USA) containing 10% fetal bovine serum (FBS; Gibco, USA), 100 U/ml penicillin, 100 μg/ml streptomycin in a 37 °C humidified incubator containing 5% CO_2_. HUVECs were cultured at 37 °C under 5% CO_2_ in medium 131 (Gibco) supplemented with 10% FBS, 5% microvascular growth supplement (MVGS; Gibco) and antibiotics as described above. *Aedesalbopictus mosquito* cells (C6/36) were grown at 28 °C with 5% CO_2_ in DMEM supplemented with 10% FBS. DENV-2 (strain TR1751), isolated from a patient with dengue fever, was a gift from Dr. A. Oya (National Institute of Infectious Disease, Japan), and was propagated in C6/36 cells grown at 28 °C in DMEM supplemented with 2% FBS, 100 U/ml penicillin, and 100 μg/ml streptomycin and then stored at −80 °C. The viral titer was detected by the plaque forming assay using monolayer culture of Vero cells under 1% methylcellulose overlay medium.

### MS analysis of the 55 kDa protein

The Coomassie blue-stained band containing 55 kDa protein isolated from ECV304 cell plasma membrane proteins was manually excised from 12% SDS-PAGE gel, minced into 1 mm^3^ pieces, and destained with washing solution [25 mM NH_4_HCO_3_, 50% (v/v) acetonitrile] until the gel slice became colorless. After freeze-drying and digesting with 10 ng/μl trypsin (Promega, USA) in 25 mM NH_4_HCO_3_ overnight at 37 °C, the gel pieces were analyzed by HPLC-(micro)chip–MS/MS (6300 ion trap system; Agilent Technologies, Santa Clara, CA). The mass spectrum was obtained, and the mass data were searched against the IPI human database (http://www.ebi.ac.uk/IPI) with the Spectrum Mill Proteomics Workbench Rev A.03.03.0 78 software (Agilent Technologies, CA)^41^,^42^.

### Characterization of the 55 kDa protein by Western blot analysis

Cell plasma membrane proteins of ECV304 cells were prepared as previously described^14^. For characterization of 55 kDa protein, total proteins, plasma membrane proteins of ECV304 cells, and excised 55 kDa protein from a gel were separated by 12% SDS-PAGE, and electrophoretically transferred onto a PVDF membrane. The mixture was placed in a blocking solution containing 5% (m/v) skim milk powder–phosphate-buffered saline (PBS) for 1 h at room temperature. The presence of the 55 kDa protein was detected by commercial rabbit anti-vimentin PcAb (1:100 dilution; Epitomics, USA) and horseradish peroxidase (HRP)-conjugated AffiniPure goat anti-rabbit IgG (1:5000 dilution; Beijing Zhongshan Golden Bridge, China) diluted in blocking solution for 1 h at room temperature. The membranes were washed six times with PBS and immersed in Amersham ECL Western blot detection reagents (GE Healthcare, UK) for 1 min. Proteins were detected by exposing the membranes by using a Bio-RAD ChemiDoc XRS processor and then photographed.

### Co-immunoprecipitation (Co-IP)

Co-IP assay of DENV-2 EDIII protein with 55 kDa protein from ECV304 cell plasma membrane proteins was performed using a co-immunoprecipitation kit (Pierce, USA). All procedures were performed on ice or at 4 °C; immunoprecipitation was performed by centrifugation. In brief, 5 μg of rabbit anti-vimentin PcAb (Epitomics) or an isotype Ab (rabbit anti-TNFα, Abcam Inc., China) was added to 50 μL of the resin slurry in a pierce spin column and incubated with agitation for 90–120 min. Approximately 10 μg of ECV304 cell plasma membrane proteins and 2 μg of the purified DENV-2 EDIII protein were added to the resin slurry-antibody mix with gentle rocking overnight at 4 °C. After three washes with IP Lysis/wash buffer, the sample was eluted by elution buffer and analyzed by 12% SDS-PAGE. The presence of DENV-2 EDIII or vimentin proteins in the sample was determined by Western blot analysis, as described above with mouse anti-DENV-2 EDIII PcAb (1:500) or commercial rabbit anti-vimentin PcAb (1:100 dilution; Epitomics), followed by secondary HRP-conjugated goat anti-mouse or goat anti-rabbit IgG antibody (1:5000, Beijing Zhongshan Golden Bridge). The membrane was finally developed with ECL (GE healthcare) and then photographed.

### Indirect immunofluorescence assay (IFA)

IFA was performed to examine the localization of DENV-2 and the superficial form of vimentin on VEC surface. HUVECs and ECV304 cells were grown on glass cover slips in 24-well plates at 37 °C with 5% CO_2_ for 18–24 h to 80% confluence. The cells were then incubated with DENV-2 (5 × 10^4^ PFU) at 4 °C for 1 h, washed with PBS, fixed with 4% paraformaldehyde, and blocked in blocking solution [1% (m/v) bovine serum albumin (BSA)–PBS] for 30 min. After washing three times with PBS, the cells were stained with mouse anti-DENV-2 EDIII PcAb (1:500 diluted in blocking solution; produced in our laboratory), followed by secondary Fluor 488-conjugated AffiniPure goat anti-mouse IgG antibody (1:400 diluted in blocking solution; Beijing Zhongshan Golden Bridge) at 4 °C for 1 h. After washing, the cells were incubated at 4 °C for 1 h with rabbit anti-vimentin PcAb (1:100; Epitomics), followed by secondary Alexa Fluor 594-conjugated AffiniPure goat anti-rabbit IgG antibody (1:400; Beijing Zhongshan Golden Bridge). Cell nuclei were stained by 4′,6-diamidino-2-phenylindole (DAPI) (Beyotime Biotechnology, China) at room temperature. After washing six times, the cells were observed under a confocal laser microscope (Leica TCS-NT, Germany). The photographs were taken and merged by the Image Proplus 5.0 program.

IFA was performed to examine the infection of DENV-2 in ECV304 cells as described previously^14^. Briefly, ECV304 cells were grown on glass cover slips to 80% confluence, and infected with DENV-2 (MOI = 1) for 0, 15, 38, and 62 h. The cover slips recovered from each time point were washed, fixed with 4% cold paraformaldehyde, and permeabilized with 0.2% Triton X-100/PBS. Nonspecific binding sites were blocked with 1% (m/v) BSA/PBS. The cover slips were incubated with mouse anti-DENV-2 EDIII PcAb (1:500) overnight at 4 °C and subsequently with a mixture of Fluor 488-conjugated AffiniPure goat anti-mouse IgG antibody (1:400) to detect DENV-2. Cell nuclei were stained by DAPI at room temperature. After washing, the cells were observed under a confocal laser microscope as previously described.

### Flow cytometric analysis

Flow cytometric analysis was performed to examine the expression level of vimentin on VECs surface and the binding characteristics of the superficial vimentin with DENV-2. ECV304 cells were re-suspended in enzyme-free cell dissociation buffer (Gibco) and incubated with DENV-2 (5 × 10^4^ PFU) at 4 °C for 1 h. After washing with staining buffer PBA (PBS containing 2% FBS and 1% sodium azide), the cells were incubated with mouse anti-DENV-2 EDIII PcAb (1:500) or homologous mouse serum as negative control for 1 h on ice, followed by Fluor 488-conjugated affinipure goat anti-mouse IgG antibody (1:400; Beijing Zhongshan Golden Bridge) for another 1 h on ice. After a final gentle wash, the cells were incubated with rabbit anti-vimentin antibody (1:50; Epitomics) and stained with Alexa Fluor 594-conjugated affinipure goat anti-rabbit IgG antibody (1:400; Beijing Zhongshan Golden Bridge) for 1 h on ice. The cells were finally analyzed by flow cytometric analysis (Partec CyFlow space, Germany), with fluorescence produced by untreated ECV304 served as background. Results were represented by means of three independent experiments ± standard deviation (S.D.).

Flow cytometry was performed to test whether the expression level of superficial vimentin would be affected by DENV-2 infection. Briefly, ECV304 cells were grown in 24-well plates at 37 °C with 5% CO_2_ for 18–24 h to 80% confluence. The cells were then infected with DENV-2 (5 × 10^4^ PFU) at 37 °C for 0, 15, 38, and 62 hpi. The infected cells were collected, stained, and subjected to flow cytometric analysis as previously described.

### Vimentin knockdown by siRNA transfection

Short interfering RNA (siRNA) duplexes (sc-29522) were purchased from Santa Cruz Biotechnology to interfere with vimentin expression. According to the manufacturer’s instructions, 1 × 10^5^ ECV304 cells in six-well culture plates were transfected with vimentin siRNA duplexes by using siRNA transfection reagent (sc-29528, Santa Cruz Biotechnology, USA). After transfection for 48 h, flow cytometric analysis was performed to determine the expression level of superficial vimentin on the ECV304 cell surface under non-permeabilized conditions at 4 °C. Normal cells served as negative control. The total vimentin protein expression levels of normal ECV304 cells and transfected cells were determined by Western blot analysis. The protein gray values were measured to evaluate interference efficiency using Image-Pro Plus 6.0 software; the gray value was relative to the protein expression level. Lysates of ECV304 cells and transfected cells were separated by 12% SDS-PAGE, and the gels were subsequently stained with Coomassie brilliant blue G-250 to confirm equal loading^43^.

### Protein expression and purification

Vimentin is composed of head, rod, and tail domains; the primers were designed to amplify these domains and the full-length vimentin (see supplementary Table 1). The total RNA of ECV304 cell was extracted by SV total RNA isolation system (Promega, USA) and then reverse transcribed to cDNA as amplification template by using reverse transcription system (Promega, USA). The expressed plasmids were constructed as previously described^44^. Briefly, the amplified PCR fragments of vimentin head and tail domains were digested by *Bam*H I/*Xho* I; and the amplified PCR fragments of full-length vimentin and vimentin rod domain were digested by *Bam*H I/ *Not* I. These fragments were then subcloned into pET22b(+) expression vector (Novagen, USA) and verified by DNA sequencing. The expression plasmids were transformed into *Escherichia coli* expression host BL21(DE3). Several single colonies were picked and grown in LB medium containing 100 μg/ml ampicillin at 37 °C with agitation. When the bacterial cultures reached an OD_600_ of 0.6, 0.5 mM isopropyl-β-D-thiogalactopyranoside (IPTG) was added to induce the expression of the target proteins for 6 h at 37 °C. The bacterial pellets were harvested by centrifugation (7,000 × *g*, 10 min) and analyzed through 12% SDS–PAGE. Clones that expressed maximal levels of the predicted recombinant proteins were chosen. The expression conditions of these clones were optimized by testing several different IPTG concentrations (0.1, 0.2, 0.5, 1.0, 2.0, and 3.0 mM), induction durations (3, 6, 12, and 18 h), and induction temperatures (25, 30, and 37 °C).

Protein expression patterns were first identified and recombinant proteins inclusion bodies were prepared to purify the above expressed proteins^43^. Briefly, 100 mL of the induced bacterial cultures were pelleted, weighed, and prepared as 10% suspension (m/v) with TBS300 buffer (100 mM NaHPO_4_, 10 mM Tris–Cl, pH 8.0, 300 mM NaCl, and 1 M urea). The suspensions were then sonicated and centrifuged. After washing three times with TBS300 containing 2% Triton X-100, the pellets were dissolved in buffer A [100 mM NaHPO_4_, 10 mM Tris–Cl, pH 8.0, 300 mM NaCl, 8 M urea, 5 mM imidazole, and 1 mM β-mercaptoethanol (β-ME)] and incubated with stirring over night at 4 °C.

For large-scale preparation of the recombinant proteins, 1 L induced bacterial cultures were collected to prepare the inclusion bodies. After centrifugation at 27, 216 × g for 20 min, the supernatant was filtered with a 0.22 μm filter unit (Millipore, USA) and captured in a Ni–NTA resin column (Bio-Rad, USA) pre-equilibrated with buffer A at a flow rate of 0.5 ml/min. The column was washed with 5 column volumes of buffer B (buffer A containing 20 mM imidazole) to remove the nonspecifically bound proteins, and the target fusion protein was eluted with buffer C (buffer A containing 150 mM imidazole). Fractions were collected and analyzed via 12% SDS–PAGE. The fractions containing the desired protein were dialyzed in refolding buffer D (100 mM NaHPO_4_, 10 mM Tris–Cl, pH 8.0, 300 mM NaCl, 4 M urea, and 1 mM β-ME), then in buffer E (100 mM NaHPO_4_, 10 mM Tris–Cl, pH 8.0, 300 mM NaCl, 2 M urea, 0.1 mM EDTA, 0.01% Triton X-100, and 10% glycerol), subsequently in buffer F (100 mM NaHPO_4_, 10 mM Tris–Cl, pH 8.0, 150 mM NaCl, 0.1 mM EDTA, 0.01% Triton X-100, and 20% glycerol), and finally in buffer G (100 mM NaHPO_4_, 10 mM Tris–Cl, pH 8.0, 150 mM NaCl, 0.1 mM EDTA, 0.01% Triton X-100, and 50% glycerol) overnight with stirring at 4 °C. The protein concentration was determined with a Bradford assay [Coomassie (Bradford) Protein Assay Kit, Pierce] and stored at −80 °C until needed.

### Vimentin-binding ELISA

To find the key domain donated to DENV adsorption, we developed a purified protein-based ELISA for testing the binding activity of each vimentin segment to DENV-2 EDIII and DENV-2. The four above-mentioned purified vimentin proteins (1 μg) were suspended in ELISA coating buffer (Beijing Dingguo Changsheng Biotechnology, China) and immobilized overnight in 96-well Immulon 2 HB plates (Thermo, USA) at 4 °C. BSA and protein-devoid systems were used as controls. The wells were washed after discarding the protein solutions. All washes were completed with 0.1 M Tris-buffered saline (TBS) at pH 7.2 with 0.05% Tween 20. Virus and antibodies were diluted by TBS; all incubations were conducted at 4 °C unless specifically stated. The wells were blocked for 1 h with 200 μL of 3% skim milk in TBS buffer and then incubated with DENV-2 (1 × 10^4^ PFU) or 1 μg of the recombinant DENV-2 EDIII protein overnight, followed by three washes and 1 h of incubation with mouse anti-DENV-2 EDIII PcAb (1:500). After washing, the wells were incubated for 1 h with peroxidase-conjugated AffiniPure goat anti-mouse IgG (1:5000) and detected by EL-ABTS Chromogenic Reagent Kit (Sangon Biotech, China). Signals were recorded at 405 nm on a VERSAmax tunable microplate reader (Molecular Devices, USA). All experiments were repeated in triplicate and reported as average and S.D.

### Infection inhibition assay and qRT-PCR analysis

To further confirm the key domain of vimentin involved in DENV adsorption, we screened four purified proteins of vimentin and a functional blocking PcAb V4630 (Sigma, USA) against the vimentin rod domain by using vimentin-binding ELISA assay and DENV entry blocking assay. For protein blocking, DENV-2 (5 × 10^4^ PFU) was pre-incubated with 50 μg/mL of the above four purified vimentin proteins, at 4 °C for 1 h. BSA was used as negative control. The virus-protein complex was then added to the HUVEC monolayer at 37 °C for 2 h. Excess or unbound virus was inactivated with acid citrate buffer (pH 3.0) and removed by extensive washing with PBS. The cells were then freeze thawed three times, and the intracellular virus titer was detected by plaque assay using Vero cells. For antibody blocking, the HUVEC monolayer was pre-incubated with anti-vimentin rod PcAb V4630 at dilutions of 1:50 and 1:100 in medium 131 at 4 °C for 1 h. Normal BALB/c mouse sera were used as negative controls. The cells were then infected with DENV-2 (MOI = 1) at 37 °C for 2 h. Excess or unbound virus was inactivated, and the intracellular virus titer was detected by plaque assay as described^8^.

Total RNA of DENV-2 infected HUVEC cells post protein- or antibody-blocking was extracted by using a TriPure isolation reagent (Roche Applied Science, USA). First strand cDNA was synthesized from total RNA (2 μg each) using RevertAid First Strand cDNA Synthesis kit (Thermo Fisher Scientific, USA) and random primers. qRT-PCR was carried out for the NS1 gene using DENV-2 serotype-specific primers^45^ and SYBR® Premix DimerEraser^TM^ (Perfect Real Time, TaKaBa, China) on a CFX connection qPCR System (BioRad, USA). The efficiency of the primer pair was determined on the basis of standard curves. Relative expression levels of NS1 gene were normalized to HUEL housekeeping gene as previously described^46^. At least three technical repeats were performed for analysis.

### Prediction of residues involved in the interaction between DENV-2 EDIII and vimentin rod

The crystallized structure of the full-length vimentin was established by MODELLER V9.7 software, and the 3D-structure databases were from Uniprot (http://www.uniprot.org/uniprot/P08670). The full-length vimentin structure was followed by docking to DENV-2 EDIII structure (PDB ID: 2JSF) using the ZDOCK V3.0.2 software to generate the docking models (14), and the best docking model was constructed after evaluating the docking complex with the pairwise shape complementarity, desolvation, and electrostatic energy methods. The interaction residues of the vimentin rod and DENV-2 EDIII proteins were predicted by the InterproSurf software, and an interacting interface was analyzed by the Pymol 1.7.3 software.

## Additional Information

**How to cite this article**: Yang, J. *et al*. Superficial vimentin mediates DENV-2 infection of vascular endothelial cells. *Sci. Rep.* **6**, 38372; doi: 10.1038/srep38372 (2016).

**Publisher's note:** Springer Nature remains neutral with regard to jurisdictional claims in published maps and institutional affiliations.

## Supplementary Material

Supplementary Dataset
