# Supplementary material for: Superficial vimentin mediates DENV-2 infection of vascular endothelial cells
Source: Sci Rep. 2016 Dec 2;6:38372. doi: 10.1038/srep38372 (PMC5133558; doi:10.1038/srep38372)
Supplement: Supplementary Dataset [file srep38372-s1.doc]

**Superficial vimentin mediates DENV-2 infection of vascular endothelial cells**

Jie Yang, Lingyun Zou, Yi Yang, Jizhen Yuan, Zhen Hu, Hui Liu, Huagang Peng, Weilong Shang, Xiaopeng Zhang, Junmin Zhu, and Xiancai Rao*

Department of Microbiology, College of Basic Medical Sciences, Third Military Medical University, Key Laboratory of Microbial Engineering under the Educational Committee in Chongqing, Chongqing 400038, People’s Republic of China

* Corresponding author mailing address: 30# Gaotanyan St., Shapingba District,

Chongqing 400038, P. R. China. Tel.: +86 23 68752240; fax: +86 23 68752240

E-mail address: raoxiancai@126.com

**Supplementary Material**

**Supplementary information contains Table and Figures**.

**Supplementary Table 1.** Primers used in this study.

| **Primer name** | **Primer sequence(5'→3’)** | **Restriction enzyme sites** | **Reference** |
| --- | --- | --- | --- |
| Vimentin head | CGGGATCCGATGTCCACCAGGTCCGTGTCCTCG | *BamH*Ⅰ | This study |
|  | CCGCTCGAGGGTGCGGGTGTTCTTGAACTC | *Xho*Ⅰ |  |
| Vimentin rod | CGGGATCCCAACGAGAAGGTGGAGCTGCAGGA | *BamH*Ⅰ | This study |
|  | CCGGCGGCCGCCCTGCTCTCCTCGCCTTCCAG | *Not*Ⅰ |  |
| Vimentin tail | CGGGATCCGATTTCTCTGCCTCTTCCAAAC | *BamH*Ⅰ | This study |
|  | CCGCTCGAGTTCAAGGTCATCGTGATGCTGA | *Xho*Ⅰ |  |
| Full-length vimentin | CGGGATCCGATTTCTCTGCCTCTTCCAAAC | *BamH*Ⅰ | This study |
|  | CCGGCGGCCGCTTCAAGGTCATCGTGATGCTGA | *Not*Ⅰ |  |

**Supplementary Figures**

**
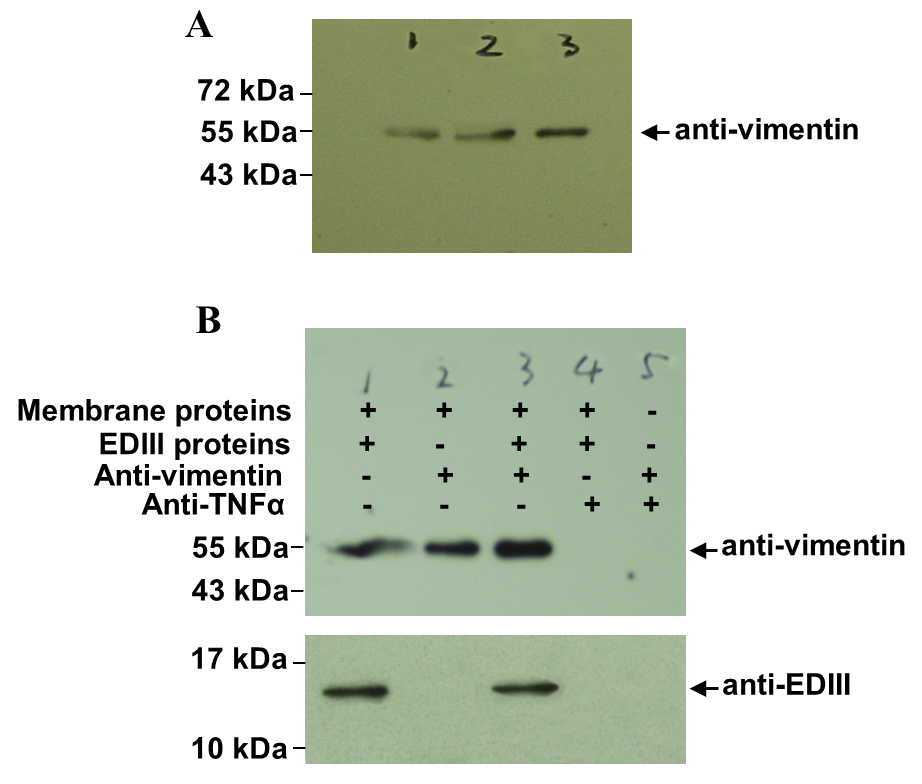
**

**Supplementary Figure 1. Characterization of the 55 kDa protein derived from the plasma membrane proteins of ECV304 cells.** (**A**) Western blot analysis of the 55 kDa protein. The total ECV304 cell proteins (lane 1), ECV304 cell plasma membrane proteins (lane 2), and the 55 kDa proteins (lane 3) excised from an SDS-PAGE gel of the plasma membrane proteins of ECV304 cells were separated by 12% SDS-PAGE and analyzed with commercial rabbit anti-vimentin PcAb as described in Materials and Methods. (B) Co-immunoprecipitation (Co-IP) experiment. The mixture of the ECV304 cell plasma membrane proteins and the purified DENV-2 EDIII was either directly analyzed (lane 1, served as protein controls) or analyzed after Co-IP assay with commercial rabbit anti-vimentin PcAb (upper panel) or mouse anti-DENV-2 EDIII PcAb (bottom panel) as indicated by an arrow on the right for testing the presence of proteins of interest (lane 3). Co-IP without DENV-2 EDIII proteins (lane 2), with an isotype Ab (anti-TNFα, lane 4) or without protenis (lane 5) served as controls. Protein sizes are indicated on the left.


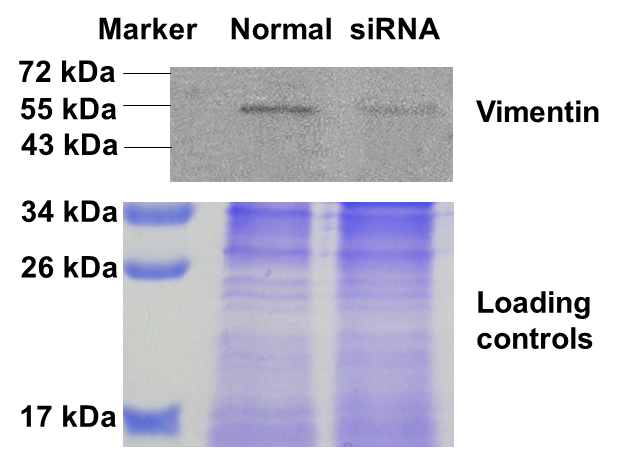


**Supplementary Figure 2.** Western blot analysis showing the knockdown efficiency of total vimentin in transfected ECV304 cells with siRNA targeting vimentin compared with untransfected normal cells. Equal loading of the gels was confirmed by staining the gel with Coomassie brilliant blue G-250. The protein sizes are indicated on the left.
